# Early postoperative endoscopic evaluation of rectal anastomoses: a prospective cross-sectional study

**DOI:** 10.1007/s00464-022-09323-6

**Published:** 2022-05-23

**Authors:** Steffen Axt, Kristin Haller, Peter Wilhelm, Claudius Falch, Peter Martus, Jonas Johannink, Jens Rolinger, Christian Beltzer, Lena Axt, Alfred Königsrainer, Andreas Kirschniak

**Affiliations:** 1 Department of General, Visceral and Transplant Surgery, Tübingen University Hospital, Hoppe-Seyler-Str. 3, 72076 Tübingen, Germany; 2 Institute of Medical Biometry, Tübingen University Hospital, Silcherstr. 5, 72076 Tübingen, Germany; 3 Department of Internal Medicine I, Hospital Reutlingen, Steinenbergstr. 31, 72764 Reutlingen, Germany; 4 General and Visceral Surgery, Maria Hilf Hospital, Viersener Str. 450, 41063 Mönchengladbach, Germany; 5 General and Visceral Surgery, Vorarlberg State Hospitals, Carl-Pedenz-Str. 2, 6900 Bregenz, Austria; 6 Department of General, Visceral and Thoracic Surgery, Federal Armed Forces Hospital Ulm, Oberer Eselsberg 40, 89081 Ulm, Germany

**Keywords:** Anastomotic leakage, Colorectal anastomosis, Flexible endoscopy, Postoperative management, Colorectal surgery

## Abstract

**Background:**

Reported incidence of anastomotic leakage (AL) of rectal anastomoses is up to 29% with an overall mortality up to 12%. Nevertheless, there is no uniform evidence-based diagnostic procedure for early detection of AL.

The objective of this prospective clinical trial was to demonstrate the diagnostic value of early postoperative flexible endoscopy for rectal anastomosis evaluation.

**Methods:**

Flexible endoscopy between 5 and 8th postoperative day was performed consecutively in 90 asymptomatic patients. Sample size calculation was made using the two-stage Simon design. Diagnostic value was measured by management change after endoscopic evaluation. Anastomoses were categorized according to a new classification. Study is registered in German Clinical Trials Register (DRKS00019217).

**Results:**

Of the 90 anastomoses, 59 (65.6%) were unsuspicious. 20 (22.2%) were suspicious with partial fibrin plaques (*n* = 15), intramural hematoma and/or local blood coagulum (*n* = 4) and ischemic area in one. 17 of these anastomoses were treated conservatively under monitoring. In three a further endoscopic re-evaluation was performed and as consequence one patient underwent endoscopic vacuum therapy. 11 (12.2%) AL were detected. Here, two could be treated conservatively under monitoring, four with endoscopic vacuum therapy and five needed revision surgery. No intervention-related adverse events occurred. A change in postoperative management was made in 31 (34.4%) patients what caused a significant improvement of diagnosis of AL (*p* < 0.001).

**Conclusions:**

Early postoperative endoscopic evaluation of rectal anastomoses is a safe procedure thus allows early detection of AL. Early treatment for suspicious anastomoses or AL could be adapted to avoid severe morbidity and mortality.

**Graphical abstract:**

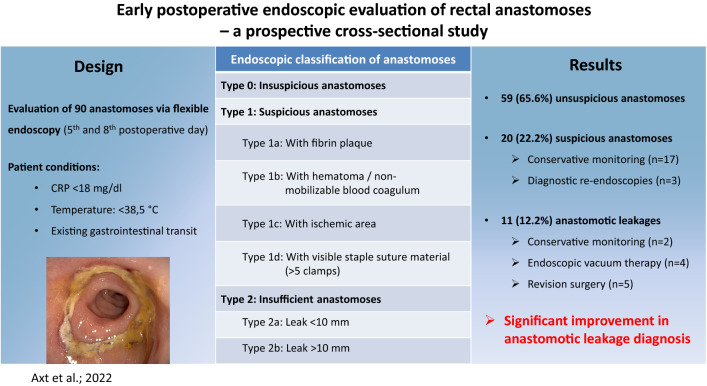

To restore intestinal continuity after rectal resections, most anastomoses are performed with the "double stapling" technique. Anastomotic leakage (AL) is one of the most relevant and common adverse events, defined as loss of intestinal wall integrity in the area of nastomosis with consecutive communication of the intra- and extra-luminal compartments [1]. The reported incidence of AL following rectal anastomosis in a systematic review of 84 prospective, partly randomized studies including 24,845 anastomoses is 11% (1–29%) and overall mortality 2% (0–12%) [2]. A recent meta-analysis with 18 prospective studies and 18,039 patients demonstrates little variation with a 9.8% AL rate [3]^.^ In a nationwide study with 577,325 patients, a consecutive mortality ("failure to rescue") of 16.4% in case of AL following colorectal surgery was reported [4].

Despite high morbidity and increased mortality risk, there is still no uniform postoperative management that avoids delayed diagnosis of AL. Late diagnosis may result in poorer outcome [5, 6].

Numerous risk factors for AL for preoperative evaluation of high-risk patients such as male gender, obesity, age > 70 years, nicotine abuse, ASA-score, preoperative radiotherapy, long duration of surgery, emergency interventions, high perioperative blood loss and not tension-free anastomoses have been determined [6–11].

To diagnose AL close clinical observation is mandatory, whereby the use of clinical scores can lead to earlier diagnosis [1]. Blood values, especially CRP, can be helpful in the follow-up [1, 6, 11, 12]. With regard to imaging procedures, computed tomography with retrograde contrast enema is widely used, but associated with a low specificity and sensitivity [13, 14]. Intraoperative endoscopy after completion of anastomosis allowed AL to be reduced [15]. However, the diagnosis of AL is commonly made in the second week after surgery [8, 10, 11]. In addition, it has already been shown that patients are diagnosed with AL even after they are discharged and that a relevant number of AL was not diagnosed until the 30th postoperative day [16–19].

Early AL (until 6 POD) were manly attributed to surgical causes and late AL to patient-related factors, which was affiliated to a poorer healing tendency [10]. This raises the question whether clinically unremarkable patients benefit from early flexible endoscopy between POD 5 and 8 following rectal anastomosis with the “double stapling” technique.

Primary endpoint of this study was a change in the postoperative therapy management after early endoscopic detection of a suspicious or insufficient anastomosis with the aim of reducing severe morbidity and mortality. Secondary endpoints were the descriptive determination of existing risk factors related to the occurrence of AL and the evaluation of a new endoscopic classification based on the endoscopic findings. Finally, a comparison with a retrospective collective in terms of morbidity, mortality, number and type of revisions and endoscopic interventions, length of hospital stay as well as treatment costs was determined.

## Materials and methods

### Patient selection

**Fig. 1:**
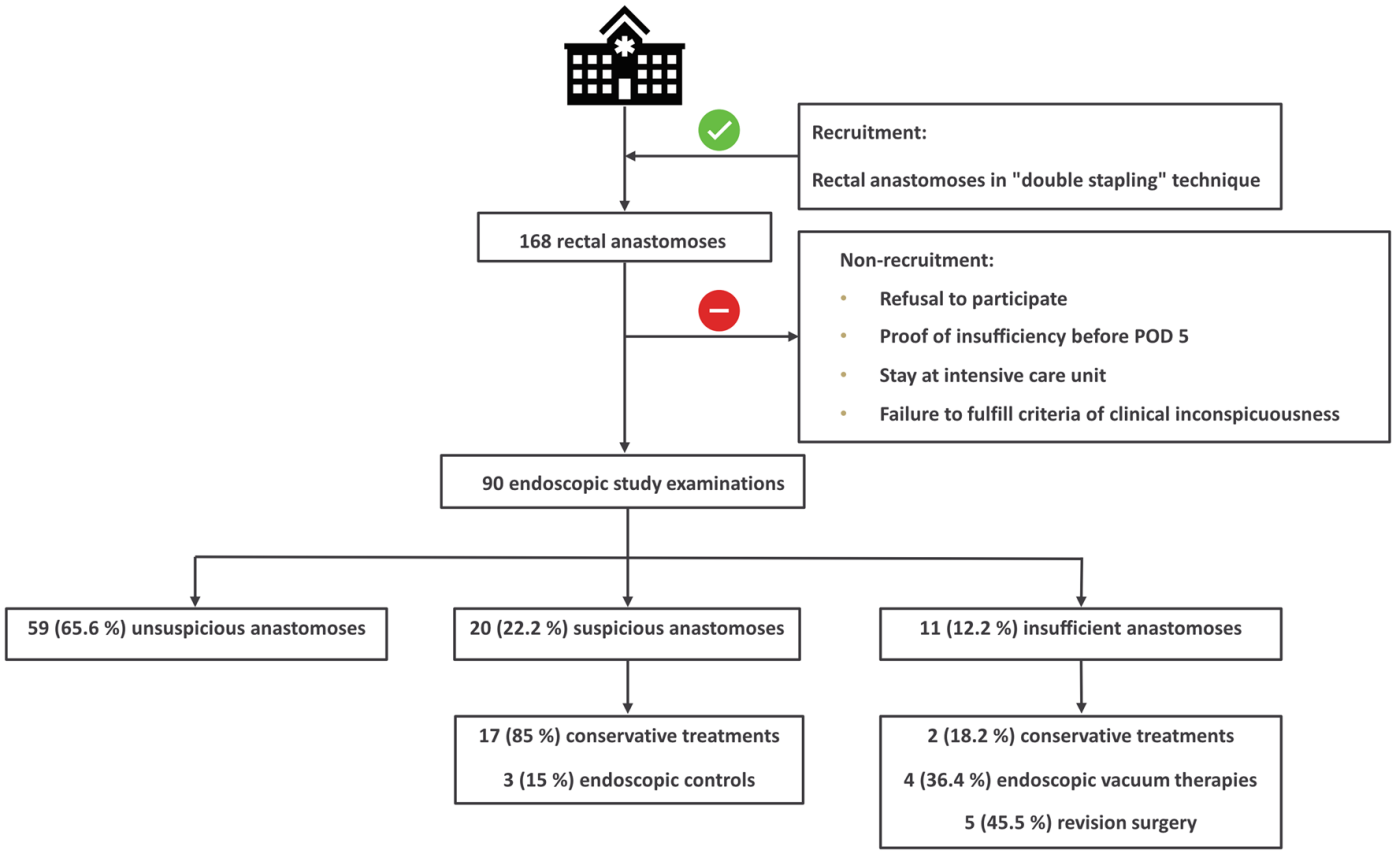
Flow chart and study examination results

In the present prospective single-center cross-sectional study, patients were recruited between 03/2018 and 03/2019 in the Department of General, Visceral and Transplant Surgery at Tübingen University Hospital, a high-volume colorectal center. A total of 90 patients out of 168 colorectal resections with performance of a rectal anastomosis created with the "double stapling" technique were consecutively recruited for the study (Fig. 1). Reasons for non-recruitment were refusal to participate, failure to fulfill the criteria of clinical unsuspiciousness, proof of AL before POD 5 or stay at the intensive care unit. Non-recruited patients constituted the retrospective comparison collective. Consecutive, a total of 78 patients received a rectal anastomosis in “double stapling” technique during the study period additionally.

### Clinical management

For elective surgery, standardized mechanical bowel preparation was performed the day before surgery using the oral laxative CitraFleet® (Recordati Pharma GmbH, Ulm, Germany) combined with intestinal decontamination by means of oral antibiotics (ciprofloxacin 500 mg and metronidazole 500 mg). No mechanical bowel preparation or oral antibiotics were used in emergency surgery. In addition, intravenous antibiotic prophylaxis with cefotaxime (2 g) and metronidazole (500 mg) was administered 1 h prior to surgery before all operations. Creation of diverting ileostomy was performed according to surgeon's valuation (decisive) taking into account neoadjuvant radiochemotherapy/radiotherapy, the distance of the anastomosis from the anocutaneous line, the reason for resection (perforation/fecal peritonitis/ischemia) and the intra-operative condition of the patient.

Study participation was approved prior to surgery and final study inclusion was determined between POD 5 and 8 after clinical evaluation. Clinically unremarkable patients with CRP < 18 mg/dl, functioning gastrointestinal transit and a body temperature ≤ 38.5 °C were then selected for early endoscopy.

Endoscopy was performed by two experienced surgical endoscopists using a flexible rectoscope (Flexible SILVER SCOPE® rectoscope n. TROIDL; Karl Storz; Tuttlingen; Germany). For standardized evaluation of the anastomoses, a new endoscopic classification was developed prior to study implementation based on a literature research and own experiences. Anastomoses were categorized as unsuspicious, suspicious or insufficient (Table 1).

**Table 1:** Endoscopic classification of anastomoses

Type 0: Unsuspicious anastomosis	Figure 2
Type 1: Suspicious anastomosis	
Type 1a: Suspicious anastomosis with fibrin plaque	Figure 3
Type 1b: Suspicious anastomosis with Hematoma/non-mobilizable blood coagulum	Figure 4
Type 1c: Suspicious anastomosis with ischemic area	Figure 5
Type 1d: Suspicious anastomosis with visible staple suture material (> 5 clamps)	
Type 2: Insufficient anastomosis	
Type 2a: Insufficient anastomosis with leak < 10 mm	Figure 6
Type 2b: Insufficient anastomosis with leak > 10 mm	Figure 7

### Statistical analysis

Data were collected from the clinical documentation systems i.s.h. med® (Siemens Medical Solutions GSD GmbH, Berlin, Germany), SAP for Healthcare® (SAP SE, Walldorf, Germany) and MEONA (Meona GmbH, Freiburg, Germany).

A two-stage Minimax Simon design was used for the primary endpoint “management change” [20]. A significance level of 0.05 (one-sided) and a power of 0.8 were chosen, an unfavorable probability of management change of *p*_0_ = 0.1 and a favorable probability of *p*_1_ = 0.2 were assumed. This resulted in a sample size of 90 patients to be analyzed. According to the Simon design, after *n*_1_ = 30 endoscopic study examinations at least four management changes had to be observed in order to continue the study and at least *r*_t_ ≥ 14 of the 90 patients had to show a management change in the final evaluation. Statistical analysis was performed using IBM® SPSS® (IBM Corporation, Armonk, New York, USA). For continuous data the *t* test and in case of non-normally distributed data the Mann–Whitney ranksum test was used. For nominal data the *χ*^2^ test and for small numbers of cases Fisher’s exact test were used. A post hoc power analysis for comparison of patients with and without a change in diagnostic management identified standardized differences of 0.63 (*T* test, independent samples) and differences in proportions of 31% could be detected with 80% power. AL risk factors were identified for standardized differences of 0.91 and differences in proportions of 43%. The level of significance was for each analysis 0.05 (two-sided) apart from the Simon design (0.05 one-sided).

### Ethics

The study was performed in accordance with the ethics requirements regarding the protection of the rights and welfare of human subjects participating in medical research (Ethics Review Board; Tübingen University; Germany: 565/2017BO2; 300/2022BO2) and complies with the criteria of the STROBE guidelines [21]. Informed consent was obtained.

The study was registered with German Clinicals Trials Register (DRKS00019217).

## Results

In total 90 clinically unremarkable patients (CRP < 18 mg/dl, body temperature < 38.5 °C, functioning gastrointestinal transit) were recruited and investigated between POD 5 and 8. Patient- and treatment-dependent characteristics are listed in Tables 2 and 3.

**Table 2:** Patient-dependent parameters

	Total [*n*]	Unsuspicious [*n*; (%)]	Suspicious [*n*; (%)]	Sufficient^X^ [*n*; (%)]	Insufficient [*n*; (%)]	*p*-value^Y^	Odds ratio^Y^	95% CI^Y^
Total	90	59 (65.6%)	20 (22.2%)	79 (87.8%)	11 (12.2%)			
Sex								
Male	48	31 (64.6)	11 (22.9)	42 (87.5)	6 (12.5)		1 (Ref)	
Female	42	28 (66.7)	9 (21.4)	37 (88.1)	5 (11.9)	0.931	1.06	0.3–3.75
Indication for surgery								
Carcinoma	54	33 (61.1)	15 (27.8)	48 (88.9)	6 (11.1)		1 (Ref)	
Benign disease	36	26 (72.2)	5 (13.9)	31 (86.1)	5 (13.9)	0.693	0.78	0.22–2.76
IBD	8	6 (75)	2 (25)	8 (100)	0 (0)		0.44	0.02–8.5
Inflammation	15	13 (86.7)	1 (6.7)	14 (93.3)	1 (6.7)		0.57	0.06–5.15
Other	13	7 (53.8)	2 (15.4)	9 (69.2)	4 (30.8)		3.56	0.83–15.18
UICC stage								
UICC 0	1	1 (100)	0 (0)	1 (100)	0 (0)		2.56	0.07–95.89
UICC I	12	5 (41.7)	6 (50)	11 (91.7)	1 (8.3)		1 (Ref)	
UICC II	6	5 (83.3)	1 (16.7)	6 (100)	0 (0)		0.59	0.02–16.68
UICC III	19	11 (57.9)	5 (26.3)	16 (84.2)	3 (15.8)		2.06	0.19–22.51
UICC IV	16	11 (68.8)	3 (18.8)	14 (87.5)	2 (12.5)	0.927	1.57	0.13–19.67
Immunosuppression								
No	81	55 (67.9)	18 (22.2)	73 (91.1)	8 (9.9)		1 (Ref)	
Yes	9	4 (44.4)	2 (22.2)	6 (66.6)	3 (33.3)	0.042	4.56	0.95–21.85
Alcohol abuse								
No	85	55 (64.7)	19 (22.4)	74 (87.1)	11 (12.9)		1 (Ref)	
Yes	5	4 (80)	1 (20)	5 (100)	0 (0)	0.391	0.59	0.03–11.38
Neoadjuvant therapy								
None	68	48 (70.6)	14 (20.6)	62 (91.2)	6 (8.8)		1 (Ref)	
Yes	22	11 (50)	6 (27.3)	17 (77.3)	5 (22.7)	0.2	3.04	0.83–11.18
RTx	2	0 (0)	1 (50)	1 (50)	1 (50)		10.3	0.57–187
CTx	9	6 (66.7)	1 (11.1)	7 (77.8)	2 (22.2)		2.95	0.5–17.5
CRTx	11	5 (45.5)	4 (36.4)	9 (81.8)	2 (18.2)		2.3	0.4–13.2
Diabetes mellitus type 2								
No	75	50 (66.7)	16 (21.3)	66 (88)	9 (12)		1 (Ref)	
Yes	15	9 (60)	4 (26.7)	13 (86.7)	2 (13.3)	0.886	1.13	0.22–5.84
Cardiovascular comorbidities								
No	42	27 (64.3)	7 (16.7)	34 (81)	8 (19)		1 (Ref)	
Yes	48	32 (66.7)	13 (27.1)	45 (83.9)	3 (6.3)	0.064	3.5	0.87–14.31
Pulmonary comorbidities								
No	79	52 (65.8)	18 (22.8)	70 (88.6)	9 (11.4)		1 (Ref)	
Yes	11	7 (63.6)	2 (18.2)	9 (81.8)	2 (18.2)	0.52	1.73	0.32–9.3
Hepatic comorbidities								
No	88	57 (64.8)	20 (22.7)	77 (87.5)	11 (12.5)		1 (Ref)	
Yes	2	2 (100)	0 (0)	2 (100)	0 (0)	0.584	1.35	0.06–29.89
Renal comorbidities								
No	84	55 (65.5)	20 (23.8)	75 (89.3)	9 (10.7)		1 (Ref)	
Yes	6	4 (66.7)	0 (0)	4 (66.7)	2 (33.3)	0.102	4.16	0.67–26.05
Smoking								
No	80	54 (67.5)	19 (23.8)	73 (91.3)	7 (8.8)		1 (Ref)	
Yes	10	5 (50)	1 (10)	6 (60)	4 (40)	0.004	6.95	1.58–30.66
ASA stage								
ASA I	3	2 (66.7)	0 (0)	2 (66.7)	1 (33.3)		1 (Ref)	
ASA II	61	43 (70.5)	13 (21.3)	46 (91.8)	5 (8.2)		4.6	0.35–60.2
ASA III	26	14 (53.8)	7 (26.9)	21 (80.8)	5 (19.2)	0.187	2.1	0.16–28.02

	Median/mean value	Maximum	IQR/SD	*p*-value
Age [years]				
Total	59	92	21	
Sufficient	60	92	19	
Insufficient	55	92	18	0.156 [T test]
Body mass index [kg/m^2^]				
Total	27.1	41.8	5	
Sufficient	26.5	39.4	4.5	
Insufficient	30.7	41.8	6.6	0.008 [T test]
Preoperative haemoglobin [g/dl]				
Total	12.7	17.6	2.1	
Sufficient	12.6	17.6	2	
Insufficient	13	15.7	2.6	0.527 [T test]
Preoperative leukocytes [1/µl]				
Total	7810	21,000	3250	
Sufficient	7950	21,000	3390	
Insufficient	6830	9570	1800	0.276 [MWU]
CRP prior to examination [mg/dl]				
Total	6.6	17.3	4.6	
Sufficient	6.3	17.3	4.5	
Insufficient	9	15.1	4.6	0.054 [MWU]

*X* sufficient anastomoses consisting of unsuspicious and suspicious anastomoses, *Y* analysis related to sufficient and insufficient anastomoses, *CI* confidence interval, *Ref* reference, *IBD* inflammatory bowel disease, *RTx* radiation therapy, *CTx* chemotherapy, *CRTx* concomitant chemoradiotherapy, *UICC* Union Internationale Contre le Cancer, *ASA* American Society of Anesthesiologists, *MWU* Mann–Whitney ranksum test

**Table 3:** Treatment-dependent parameters

	Total [*n*]	Unsuspicious [*n*; (%)]	Suspicious [*n*; (%)]	Sufficient^X^ [*n*; (%)]	Insufficient [*n*; (%)]	*p*-value^Y^	Odds ratio^Y^	95% CI^Y^
Total	90	59 (65.6%)	20 (22.2%)	79 (87.8%)	11 (12.2%)			
Time of surgery								
Elective surgery	83	55 (66.2)	17 (20.5)	72 (86.7)	11 (13.3)		1 (Ref)	
Emergency surgery	7	4 (57.1)	3 (42.9)	7 (100)	0 (0)	0.304	2.38	0.13–44.5
Surgery access								
Laparoscopic	39	30 (76.9)	6 (15.4)	36 (92.3)	3 (7.7)		1 (Ref)	
Open	28	20 (71.4)	5 (17.9	25 (89.3)	3 (10.7)		1.44	0.27–7.72
Robotic	23	9 (39.1)	9 (39.1)	18 (78.2)	5 (21.8)	0.253	3.33	0.72–15.54
Level of anastomosis								
> 10 cm ab ano	46	34 (73.9)	7 (15.2)	41 (89.1)	5 (10.9)		1 (Ref)	
10–5 cm ab ano	21	13 (61.9)	6 (28.6)	19 (90.5)	2 (9.5)		0.86	0.15–4.85
< 5 cm ab ano	13	12 (52.9)	7 (30.4)	19 (82.6)	4 (17.4)	0.672	2	0.33–12.25
Expansion of surgery								
No	79	50 (63.3)	20 (25.3)	70 (88.6)	9 (11.4)		1 (Ref)	
Expansion	11	9 (81.8)	0 (0)	9 (81.8)	2 (18.2)	0.52	1.73	0.33–9.3
Intraoperative transfusion								
No	85	55 (64.7)	19 (22.4)	74 (87.1)	11 (12.9)		1 (Ref)	
Yes	5	4 (80)	1 (20)	5 (100)	0 (0)	0.391	1.7	0.09–32.8

	Median/mean value	Minimum	Maximum	IQR/SD	*p*-value
Duration of surgery [minutes]					
Total	218	70	511	88	
Sufficient	214	70	511	87	
Insufficient	245	120	419	94	0.368 [MWU]
Intraoperative volume substitution [ml]					
Total	3250	20	12,980	2520	
Sufficient	3100	0	11,300	2400	
Insufficient	4500	1000	13,000	3500	0.220 [MWU]
Intraoperative blood loss [ml]					
Total	199	0	2310	363	
Sufficient	200	0	2300	365	
Insufficient	180	0	1200	360	0.782 [MWU]

*X* sufficient anastomoses consisting of unsuspicious and suspicious anastomoses, *Y* analysis related to sufficient and insufficient anastomoses, *CI* confidence interval, *R* Reference, *IBD* Inflammatory bowel disease, *RTx* radiation therapy, *CTx* chemotherapy, *CRTx* concomitant chemoradiotherapy, *UICC* Union Internationale Contre le Cancer, *ASA* American Society of Anesthesiologists, *MWU* Mann–Whitney ranksum test

**Fig. 2:**
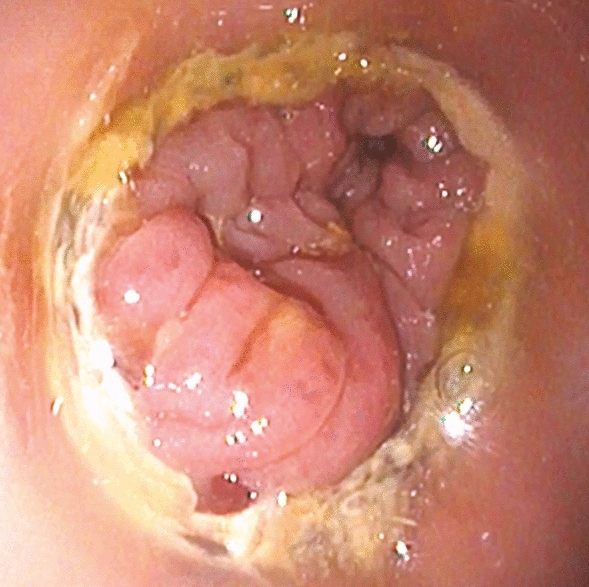
Unsuspicious anastomosis (Type 0)

**Fig. 3:**
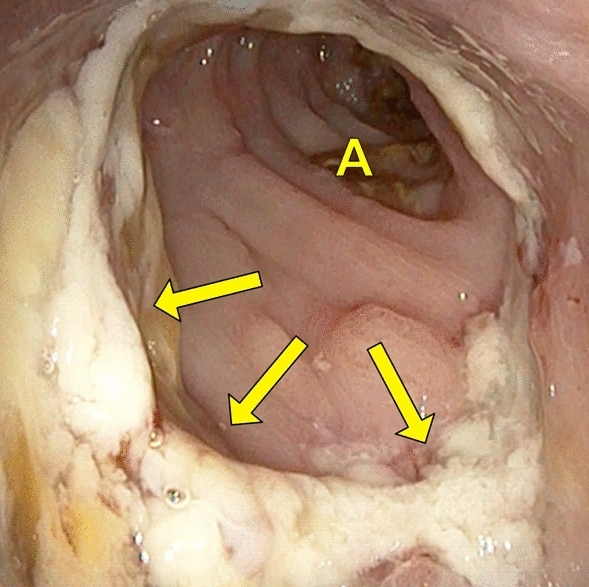
Suspicious anastomosis with fibrin plaque (Type 1a); A: Intestinal lumen; Arrow: Fibrin plaque

**Fig. 4:**
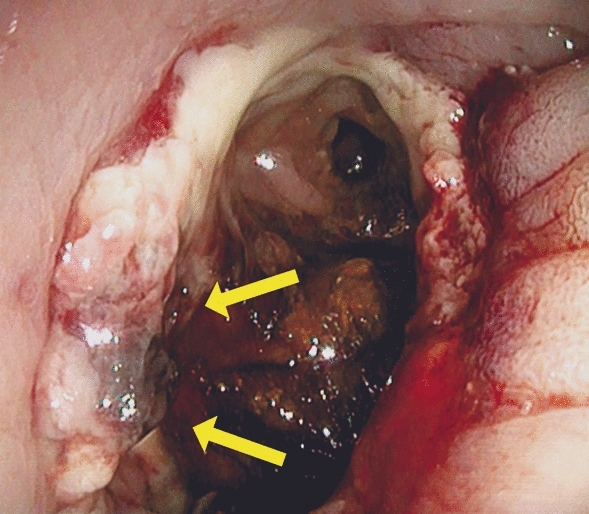
Suspicious anastomosis with Hematoma/non-mobilizable blood coagulum (Type 1b); Arrow: Non-mobilizable blood coagulum

**Fig. 5:**
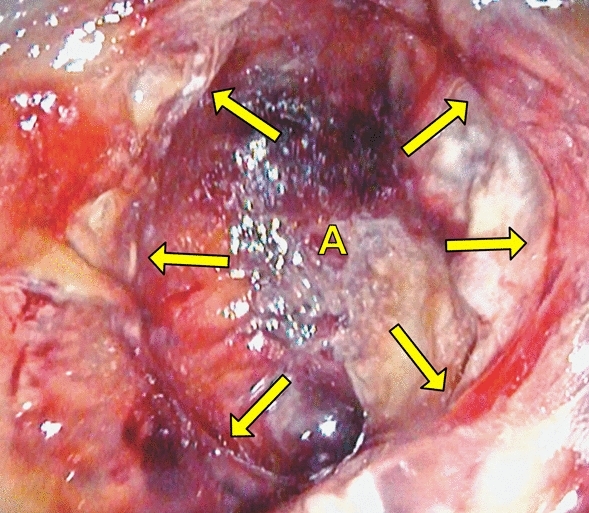
Suspicious anastomosis with ischemic area (Type 1c); A: Intestinal lumen; Arrow: Anastomosis

The endoscopic classification (Table 1) showed 59 (65.6%) anastomoses to be unsuspicious with proper healing (Fig. 2) and 20 (22.2%) to be suspicious with delayed healing. Here, 15 (16.7%) anastomoses had partial fibrin plaques (Fig. 3), four (4.4%) a hematoma or non-mobilizable blood coagulum (Fig. 4) and one (1.1%) an ischemic area in the region of anastomotic connection (Fig. 5). None of the anastomoses showed staple suture material (> 5 clamps). In the 20 patients with suspicious anastomosis hospital stay after study endoscopy was prolonged with or without antibiotic therapy in 17 patients. With regredient blood values of inflammatory markers and lack of clinical aggravation, no further intervention was performed in these study participants. In one of these cases, re-laparotomy was performed for existing ileus and abdominal wall abscess. Here, too, no evidence for AL was detected intraoperatively. A scheduled re-endoscopy was performed in three. In one case, the diagnostic re-endoscopy revealed a persistently suspicious anastomosis without regression of CRP. Consequently, endoscopic vacuum therapy (EVT) was performed. Later, re-laparotomy for incarcerated parastomal hernia with no intra-operative evidence of AL was indicated. EVT was able to be completed after healing of the anastomosis. The other 2 re-endoscopies did not detect any worsening of the anastomoses, so that no further intervention was necessary. In total, none of the suspicious anastomoses became insufficient during the further course and no re-laparotomy caused by AL was needed.

**Fig. 6:**
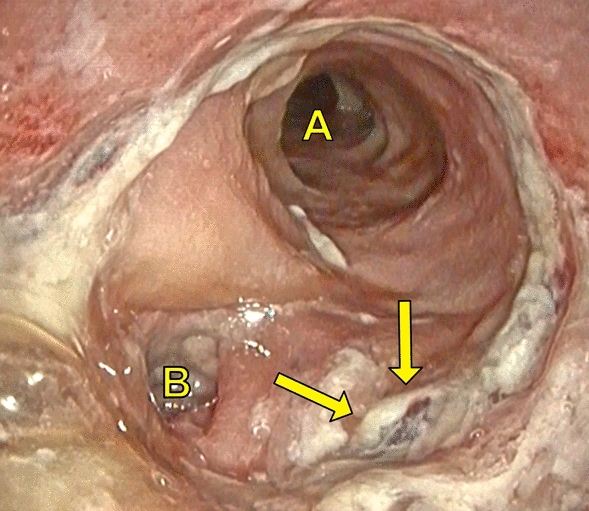
Insufficient anastomosis with leak < 10 mm (Type 2a); A: Intestinal lumen; B: “Blind branch”; Arrow: Anastomotic leak (< 10 mm)

**Fig. 7:**
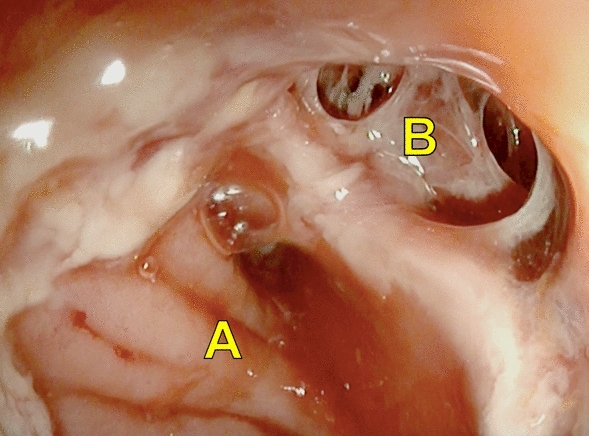
Insufficient anastomosis with leak > 10 mm (Type 2b); A: Intestinal lumen; B: Anastomotic leak (> 10 mm)

In 11 (12.2%) patients AL was detected, seven (7.8%) were < 10 mm (Fig. 6) and four (4.4%) > 10 mm (Fig. 7). In two (2.2%) patients diverting ileostomy the AL was small in size without evidence of an extra-luminal cavity. In addition to close clinical and blood count monitoring antibiotic treatment was performed and no further endoscopic intervention or surgery was necessary. Four patients with AL successfully underwent EVT with complete resolution of the anastomosis. No persistent AL was observed and no patient had to be operated. Five (5.6%) patients with AL needed revision surgery, namely a Hartmann procedure in two patients, while another two patients received a diverting stoma (1 transversostomy; 1 ileostomy). In one patient a new anastomosis without diverting ileostomy was created. An overview of the findings of the endoscopic examination and the resulting management is shown in Fig. 1.

According to the two-stage Simon design, the performed endoscopic study examination had to result in a minimum number of *r*_t_ ≥ 14 consecutive changes in therapy management. The examinations revealed a total of 31 (34.4%) changes in therapy management. The result was significant in the Simon design (31 vs. 14, 95% CI 0.25–0.45; *p* < 0.0001, Binomial test, not adjusted for the Simon design). This results in a statistically significant improvement in AL diagnosis based on an early postoperative endoscopy between POD 5 and 8.

In the comparison collective 65 (83.3%) patients showed no evidence of AL. Here, 4 revisions unrelated to the anastomosis (e.g., due to hernia, ileus, etc.) were performed. Furthermore, 4 diagnostic endoscopies became necessary, without proof of AL.

In 11 (14.1%) patients, AL could be detected during hospital stay. Diagnosis was made by endoscopy in 6 cases and by computed tomography in 5 cases. Surgical revision was necessary in 8 cases, of which a new anastomosis was created in 4 cases and a Hartmann procedure was made in 4 cases. Endoscopic vacuum therapy was performed once. One patient was treated conservatively and one died directly after diagnosis of AL on POD 6. Overall mortality was 3 (3.8%), with cardiac events as another cause.

Risk factors showed there was a significant difference in AL rate between smokers (*n* = 4; 40%) and non-smokers (*n* = 7; 8.8%) (*p* = 0.016). Moreover, a significant difference was also detected with regard to body weight. Mean BMI of patients without AL was 26.5 (SD 4.5) kg/m^2^ and in those with AL it was 30.7 (SD 6.6) kg/m^2^ (*p* = 0.008). The ordinal logistic regression analysis pointed to a significantly increased risk (*p* = 0.001) for AL in obese patients with nicotine abuse. Calculation of the receiver operating characteristic curve yields a positive predictive value of 80.7% for AL in overweight smokers. Furthermore, a significant increase in AL was found in immunosuppressed patients (*n* = 9; 10%), 33.3% versus 9.9% corresponds with p = 0.042. The results of patient-related and treatment-related characteristics are listed in Tables 2 and 3.

No further significant differences in the occurrence of AL were determined using CRP alone as marker. Mean CRP on the day before endoscopy was 6.3 (SD 4.5) mg/dl for non-insufficient and 9 (SD 4.6) mg/dl for insufficient anastomoses.

A comparison of 38 patients with an ileostomy and 52 without revealed no significant difference in AL rate (*p* = 0.286) (Table 4).

**Table 4:** Analyses of diverting stomata, length of hospital stay and treatment costs

	Total	Unsuspicious	Suspicious	Insufficient	*p*-value^y^
Diverting stoma [*n*; (%)]					
No	52 (100)	41 (78.9)	6 (11.5)	5 (9.6)	
Yes	38 (100)	18 (47.4)	14 (36.8)	6 (15.8)	0.286
Length of hospital stay [days]					
Total		9	10	29	0.002
After study endoscopy					
No intervention/surgery		–	4	13	
EVT		–	–	24	
Revision surgery		–	36	14	
Treatment costs [€]		13.017	15.820	26.192	0.002

*EVT* endoscopic vacuum therapy, *Y* Analysis related to sufficient (unsuspicious and suspicious) and insufficient anastomoses

Average hospital stay in the study collective of patients with unsuspicious anastomoses was 9 days, with suspicious anastomoses 10 days and with AL 29 days (Table 4). In the comparison collective, length of hospital stay of patients without evidence of AL was 10 days and with AL 22 days. In both, the study and the comparison collective, there was a significant increase in the length of hospital stay when AL was detected (*p* = 0.002 and *p* = 0.001). The comparison of the collectives demonstrated no significant difference for both sufficient anastomoses (*p* = 0.672) and AL (*p* = 0.676). The average treatment costs in the study collective in case of unsuspicious anastomoses were €13.017, in case of suspicious anastomoses €15.819 and with diagnosed AL €26.192. A significant difference in these costs between patients with sufficient compared to insufficient anastomoses could be demonstrated (*p* = 0.002). In the comparison collective, treatment costs for patients without evidence of AL were €16.051 and for patients with diagnosed AL €31.259. This resulted in a significant difference in treatment costs (*p* = 0.001). There were no significant differences between patients with sufficient anastomoses (*p* = 0.833) and insufficient anastomoses (*p* = 0.849) when comparing the study and the comparison collective.

## Discussion

An AL rate up to 29% and overall mortality up to 12% following colorectal resection have been reported [2]. Symptoms of AL vary in their severity and are difficult to define from postoperative sequelae or other postoperative adverse events, thus making AL diagnosis demanding, especially in patients with a diverting stoma. Clinical assessment of AL prediction following surgery with a sensitivity of 62% and a specificity of 52% remains unsatisfactory [22].

Recent data confirm a positive influence of intra-operative endoscopy for evaluation of anastomoses. A meta-analysis of 6 studies with *n* = 1.084 patients showed a significant reduction of AL rate from 6.9 to 3.5% (OR 0.37; 95% CI 0.21–0.68; *p* = 0.001) [15]. Intraoperative endoscopy is a simple and reliable diagnostic tool. However, a regular intra-operative anastomosis does not preempt an insufficiency occurring in the postoperative course [23]. The period of occurrence of AL is mainly reported as 1–2 weeks postoperatively [8, 18, 24]. A subdivision into early AL until POD 6 and late AL was undertaken, defined as occurrence from POD 6 [10] to 30 or following hospital discharge [19, 25–28]. Causes of early AL include mainly surgical technical problems, while patient- or tissue-related factors are causes of late AL [10, 11]. Diagnosis of AL after POD 30 with an incidence of up to 42% is quite high and asymptomatic in most patients [11, 18, 19].

Currently, computer tomography is the most frequently used diagnostic tool for clarification of AL. For use of water-soluble contrast enema, a sensitivity of 52.2–83.3% and a specificity of 78–100% is declared [6, 13, 14]. Contrast enema extravasation is the most reliable sign for AL detection whereas only 15–17% can be proven [13]. Marres et al. demonstrated a significantly higher mortality and a significantly longer hospital stay due to the therapy delay caused by false-negative CT diagnosis with a positive predictive value of 78% (95% CI 0.65–0.92) and a negative predictive value of 88% (95% CI 0.82–0.95) in the false-negative group [5].

Delayed diagnosis of AL is associated with poorer patient outcome, namely with a poor functional result, a higher mortality rate and also a poorer oncological outcome. The local recurrence rate is higher and distant metastases occur more frequently [2–4, 6, 29, 30]. It is astonishing that despite these options there is no standardized diagnostic algorithm or procedure for suspected AL.

Primary endpoint of this study was a change in the postoperative therapy management after early endoscopic detection of a suspicious or insufficient anastomosis in order to avoid severe morbidity and mortality. A total of 31 (34.4%) changes in postoperative management caused by AL in 11 (12.2%) and suspicious anastomosis in 20 (22.2%) patients were the consequence of early endoscopy. This results in a statistically significant improvement (0.35; 95% CI 0.25–0.46; *p* = 0.006) in early diagnosis of AL between POD 5 and 8 and therefore in postoperative management due to early endoscopic control of rectal anastomoses.

Of the 20 patients with suspicious anastomoses 17 were treated successfully with exclusively conservative means. Indication for re-endoscopy was only given in 3 patients, which resulted in requirement for EVT in one patient. For other reasons, two surgical revisions had to be performed, in which intra-operative findings also demonstrated no evidence of AL. This demonstrates that conspicuous anastomoses in this study were mostly in need of further monitoring only. In 11 patients without symptoms AL was detected during the early endoscopic examination. It can therefore be assumed that AL was diagnosed early and that more serious adverse events were avoided. Despite the fact that an AL rate of 12.2% in unremarkable patients appears to be high, there were zero mortalities. As a consequence of endoscopy, management was immediately adjusted and patients received appropriate treatment (5 surgical revisions and 4 endoscopic interventions). Furthermore, two patients with a small AL and a diverting ileostomy were successfully treated conservatively.

The retrospective comparison collective revealed an AL rate of 14.1% and a mortality rate of 3.8% which were thus slightly higher than in the study population. As a result of this, 8 surgical revisions, with definitive treatment using Hartmann procedure in 4 cases, were performed. In contrast, only 5 surgical revisions with only 2 Hartmann procedures, but 4 successful endoscopic and 2 conservative therapies were conducted in the study collective. This results in a not significant increased length of hospital stay for patients with AL of 29 days in the study population compared to 22 days in the comparison collective (*p* = 0.676). In terms of treatment costs, there was also no significant difference between the two collectives in the presence of AL (*p* = 0.849), with costs being on average €5.000 (€31.259 vs. €26.192) less in the study population. Thus, it can be assumed that early diagnosis of AL with consecutive possibility of therapy adaptation increases the likelihood of endoscopic or less invasive surgical therapies. This in turn does not lead to any rise in the length of hospital stay or treatment costs.

A statistically significant accumulation of smokers, obese and immunosuppressed patients were found in this collective. Smoking (40% vs. 8.8%; *p* = 0.016), overweight (30.7 vs. 26.5 kg/m^2^; *p* = 0.008) and immunosuppression (33.3% vs. 9.9%; *p* = 0.042) were significant risk factors for AL, as already proved by other studies [6, 7, 9, 10, 31]. CRP was also found to be almost significantly increased (*p* = 0.054) in patients with proven AL compared to patients without AL. Post-operative CRP was described as a good predictive value for AL (89–97%) [6, 12, 32, 33].

Vallicelli et al. retrospectively evaluated 52 patients who underwent fluorescence angiography with fluorophore indocyanine green and flexible endoscopy during colorectal anastomosis. 12 anastomotic defects (insufficiency, mucosal crash, edema or bleeding) were detected and corrected with immediate suture reinforcement. Of these, none proved to be insufficient in the further course. However, insufficiencies occurred postoperatively in 3 patients whose anastomoses were unsuspicious intraoperatively. Also, the classification used only includes acute conditions in the anastomosis evaluation. Patient-dependent healing defects of anastomoses such as increased fibrin deposition in the course are not taken into account in this classification. Therefore, the classification of Vallicelli et al. is not applicable to postoperative anastomosis evaluation [34].

Sato et al. also retrospectively classified 80 anastomoses before ileostomy reversal about 6 weeks after surgery and correlated the results with the functional outcome. The classification took erythema, erosion, ulceration, granulomatous change, fine granular protrusions, white-coated or hemorrhagic mucosa into account and did not include insufficiencies. Therefore, there is no applicability of this classification for postoperative anastomosis control as indicated by our classification [35].

A certain limitation of this study could be the heterogeneous collective, but the anastomoses were performed with the same technique regardless of the operation performed. The sample size was statistically determined for the primary endpoint, so that the possible effects of risk factors could only be done descriptively. Endoscopic evaluation of anastomoses with this new classification appears to be precise and allows uniform classification and individual therapy. In addition, we can speculate that severe adverse events were able to be avoided.

In our study, 31 (34.4%) of the 90 clinically unremarkable patients were seen to have a suspicious anastomosis (*n* = 20; 22.2%) or AL (*n* = 11; 12.2%). A total of 40 (43.3%) patients had a diverting stoma. It was noticeable that diverting stomata were more frequent in patients with suspicious anastomoses (15 out of 20) and in six of 11 patients with AL. Therefore, these data lead us to assume a large number of undiagnosed suspicious anastomoses and AL in patients with a diverting stoma. A retrospective analysis of 395 anastomoses following rectal cancer surgery revealed 8.1% with AL. In 22% of the cases, AL was diagnosed after POD 60 [36]. These patients had a large number of diverting ileostomies and the need for definitive stoma creation was significantly greater than in our cohort. A further retrospective analysis involving 998 patients following low anterior resection revealed an AL rate of 20%, with 33% of these diagnosed after POD 30. AL until POD 30 in patients without diverting stoma was significantly more frequent than in patients with diverting stoma (19.2% vs. 11.4%; *p* < 0.01). In cases of proven AL mortality increased from 1% until POD 30 to 3% until POD 90 [37]. Anastomoses with diverting ileostomies should therefore be extensively clarified postoperatively.

Early endoscopic evaluation of rectal anastomoses between POD 5 to 8 is an effective means of assessing anastomotic healing with the option of avoiding delayed diagnosis of AL. Known risk factors permit identification of patients with a high-risk anastomosis and therefore with a high risk for AL in order to select patients for early endoscopy. Endoscopy with a standardized endoscopic classification therefore con improve postoperative management with a probability of better patient outcome. In addition, early diagnosis of AL may prevent the need for revision surgery by allowing early EVT. Further studies, especially randomized controlled trials, should examine the integration of early endoscopic evaluation of rectal anastomoses with a standardized endoscopic classification into postoperative management.
